# Eco-Friendly Ether and Ester-Urethane Prepolymer: Structure, Processing and Properties

**DOI:** 10.3390/ijms222212207

**Published:** 2021-11-11

**Authors:** Joanna Niesiobędzka, Ewa Głowińska, Janusz Datta

**Affiliations:** Department of Polymer Technology, Faculty of Chemistry, Gdańsk University of Technology, G. Narutowicza St. 11/12, 80-233 Gdańsk, Poland; joanna.niesiobedzka@pg.edu.pl (J.N.); janusz.datta@pg.edu.pl (J.D.)

**Keywords:** ether-urethane prepolymer, ester-urethane prepolymer, bio-based diisocyanate, chemical structure, thermal analysis, processing properties

## Abstract

This study concerns bio-based urethane prepolymers. The relationship between the chemical structure and the thermal and processing parameters of bio-based isocyanate-terminated ether and ester-urethane prepolymers was investigated. Bio-based prepolymers were obtained with the use of bio-monomers such as bio-based diisocyanate, bio-based polyether polyol or polyester polyols. In addition to their composition, the bio-based prepolymers were different in the content of iso-cyanate groups content (ca. 6 and 8%). The process of pre-polymerization and the obtained bio-based prepolymers were analyzed by determining the content of unreacted NCO groups, Fourier transform infrared spectroscopy, proton nuclear magnetic resonance, thermogravimetry, and rheological measurements. The research conducted facilitated the evaluation of the properties and processability of urethane prepolymers based on natural components. The results indicate that a significant impact on the processability has the origin the polyol ingredient as well as the NCO content. The thermal stability of all of the prepolymers is similar. A prepolymer based on a poly-ether polyol is characterized by a lower viscosity at a lower temperature than the prepolymer based on a polyester polyol. The viscosity value depends on the NCO content.

## 1. Introduction

For the first time, Bayer obtained polyurethanes in 1947 and since then the interest in them has been growing steadily [1,2]. Chemical industry products, including polyurethanes, help to reduce energy costs and the negative environmental impact of the industry. Plastics-made car parts significantly reduce vehicle weight, which has a positive effect on fuel consumption. This improvement has been estimated at around 7% for every 10% reduction in vehicle weight. Moreover, a report from the American Chemistry Council shows that the use of appropriate polymer materials in buildings can save 40 times the energy used to produce it. It was also found that over the years plastics and insulation materials have helped to save 467.2 trillion BTU (BTU—British thermal unit) [3].

Polyurethanes can be a beneficial and safe way to reduce carbon dioxide emissions and the effects of global warming. The demand for these plastics is steadily increasing due to reduced production losses, increased recyclability, and the extended life cycle of the end product caused by advancements in polyurethanes materials manufacturing technology.

Federal governments and agencies are increasingly concerned about the harmful health effects of isocyanates used in polyurethane production. In addition, the production of polyols, which are the second essential component of plastics, depends on the availability of fossil fuels. Their production process is also environmentally harmful. Consequently, the polyurethane industry is constantly striving to sustainably develop its business practices. According to the Global Risks Report prepared in 2020, an unstable geopolitical situation in oil extraction regions contributed to a price shock, which additionally translated into instability on the dependent market of raw materials [4]. Over the past year, oil prices have risen by 158.71% on the stock market [5]. By comparison, the price of corn (the input for bio-glycols production), which can be processed into raw materials necessary for the production of polyurethanes, has increased by 60.75% [6]. Such price fluctuations make natural resources a more stable source for the production of urethane monomers. Moreover, for the first time in 2019, among the members of the World Economic Forum community, environmental concerns dominated the top long-term risks by likelihood of occurrence. Among the top global risks were listed: failure of climate change mitigation and adaptation by government and business, and human-made environmental damage and disasters [4]. Due to the above mentioned aspects, it is important to develop substrates from natural resources for use in polyurethane production.

Polyurethanes can be obtained by a two-step method which involves the use of prepolymers [7]. In the first step, the polyol reacts with a moderate excess of the diisocyanate relative to the hydroxyl groups of the polyol. The isocyanate is attached to the end groups of the polyol to form a product containing terminated isocyanate groups and an excess of diisocyanate. The resulting prepolymer contains a certain percentage of free isocyanate groups, on the basis of which the amount of the chain extender to be added is calculated. In the second step, the prepolymer is reacted with a predetermined amount of the chain extender in the presence of a catalyst [8].

The use of excess isocyanate is required to prevent the increase in molecular weight caused by the chain length increase in the unreacted polyol, reacting with the prepolymer with free isocyanate groups. Prepolymers in which excess isocyanate remains in the product are called “quasi-prepolymer”. In practice, this term refers to materials with high levels of unreacted isocyanate (>12%), while the term “prepolymer” is used for low levels of unbound isocyanate (<12%).

Among the numerous advantages of prepolymers, particular attention should be paid to their stability during storage and ease of processing because of their aggregate state (liquid). Furthermore, the prepolymer ensures better compatibility with additional components of the mixture and allows for a more homogeneous structure of the final product than those obtained in one-step synthesis [9]. A very important advantage of prepolymers is the ability to pre-build the properties of the polymer. A number of these advantages influence the high demand for prepolymers, which is approximately 500 million pounds per year for commercial applications [10]. Isocyanate-terminated urethane prepolymers are the intermediate in the polyurethane preparation with the prepolymers method, and can be processed with industrial grade machines. Based on previous studies, it was found that prepolymers containing 6 to 8% unreacted NCO groups have high application potential due to their viscosity. Kasprzyk et al. proved that an increase in the content of unreacted NCO groups contributes to an increase in the degree of phase separation, because of the higher content of hydrogen bonds between urethane-urethane groups. It was also found that the materials obtained from the prepolymer containing 8.0% of unreacted NCO groups showed the highest content of rigid segments. The content of rigid segments affects the value of tensile strength and Young’s modulus [11].

In industrial processes, one of the most important processing properties of polyurethanes is their rheological behavior of components. Rheological measurements allow for determination of the viscosity of fluids under specific conditions of pressure and temperature. Rheological behavior and viscosity are also related to the structure of polymer chains [12].

Rheological property studies are important for:

- designing pipelines, processing equipment, pumps, etc.;
- the quality control of intermediate and end products;
- the analysis of properties of substances during their use.

In developed countries, the total amount of processed polyurethanes is several million tons. Therefore, even small improvements to processing properties result in positive economic changes. Research on rheological measurements is also essential in terms of industrial modernization and increasing demand for high quality of end products.

Current scientific work focuses on transforming industry to become more ecological, e.g., by using raw materials of biological origin [13]. According to European standard EN16575, bio-based products are defined as partially or completely derived from biomass. They are important elements of the implementation of the bio-economy, in which products of biological origin replace those of the mining industry. The bio-economy sector, located only in the European Union, is estimated to generate a turnover of EUR 2.1 billion and create jobs for more than 18 million people [14]. Fossil fuel mining can pose a threat to the environment, so effective measures to minimize negative environmental impacts are now a priority for future generations. An additional problem related to the extractive industry is growing prices of petroleum products and their limited resources [15]. Thus, the use of renewable raw materials that are available and relatively inexpensive is a desired alternative. The benefits of processing bio-monomers are also an important advantage of natural raw materials, they reduce energy consumption by lowering the activation energy of polyurethane processing [12].

Due to the considerable interest in materials of natural origin and the policy of sustainable development, it is expected that there will be an increase in the production of bio-monomers as components in the synthesis of polyurethane materials. Bio-monomers for the polyurethane industry are produced from plant biomass. There are several manufacturers in the market offering commercially available biopolyols. One of them is Velvetol produced by Allessa [16] or PriplastTM 3238 offered by Croda [17]. The market of commercially available bio-isocyanates, however, is poorer than the market of bio-polyols. There are three major producers of bio-isocyanates. One of them is Covestro, which offers a PDI trimer under the trade name Desmodur^®^ eco N 7300 [18]. Mitsui Chemicals offers bio-diisocyanate under the trade name STABIO™ PDI [19]. Another manufacturer of bio-isocyanates is Vencorex, which offers Tolonate™ X FLO 100 [20].

In the present study, the structure-property relationships of novel bio-based urethane prepolymers prepared with bio-monomers were investigated. We studied the correlation between chemical structure of the obtained bio-based urethane prepolymers and their thermal and rheological properties. To the best of our knowledge, there is no detailed publication on the thermal properties of prepolymers derived from bio-based raw materials.

## 2. Materials and Methods

### 2.1. Materials

In the synthesis of prepolymers based on renewable raw materials we used aliphatic isocyanate Tolonate X FLO 100 provided by Vencorex (Saint-Priest, France), two types of polyols: PRIPLAST 3294^TM^ (Priplast) semicrystalline polyester bio-polyol supplied by Croda (Snaith, UK) and Velvetol H2400 (PO3G) polyether bio-polyol supplied by Allessa GmbH (Frankfurt, Germany). The reaction inhibitor was orthophosphoric acid supplied by POCH Gliwice (Gliwice, Poland).

### 2.2. Prepolymer Preparation

**Scheme 1:**
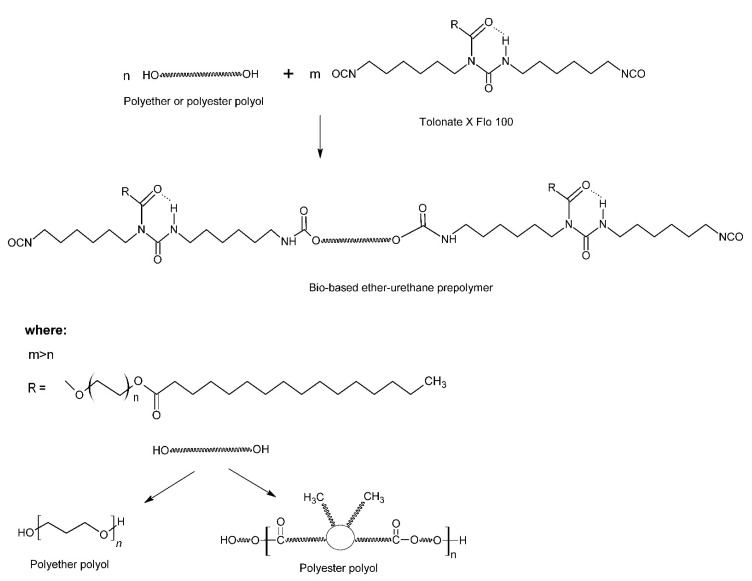
Reaction scheme for ether- and ester-urethane prepolymers formation.

The research involved obtaining ether-urethane and ester-urethane prepolymers with ca. 6% and 8% unreacted NCO content. The reactor placed in the heating jacket was filled with polyol. A thermometer and a mechanical stirrer were placed in the lid of the reactor. Initially, the polyol was heated to 60 °C and then degassed under vacuum at 90–95 °C for 1.5 h. In the next step, the mixture was cooled to 60 °C and the isocyanate and the reaction inhibitor were added. The inhibitor was used because it inhibits the formation of cross-linking bonds to prevent excessive increase in the viscosity of the prepolymer. The synthesis was carried out at the temperature of 85 °C for another two hours. A scheme of laboratory apparatus for the synthesis of urethane prepolymers is included in the Supplementary Materials as Figure S1. During the reaction, the percentages of NCO groups were determined by titration according to ISO 14896:2010. Table 1 shows the actual amounts of free isocyanate groups measured after the two-hour reaction time. Scheme 1 shows the overall ether and ester-urethane synthesis method. The structure of Tolonate X FLO 100 in the Scheme 1 was adopted from [21].

### 2.3. Characterization

#### 2.3.1. Fourier-Transform Infrared Spectroscopy (FTIR)

The infrared spectra of the polyether urethane prepolymers, polyester urethane prepolymers, and monomers were obtained with a Nicolet 8700 spectrophotometer. The spectra were recorded after 64 scans in a wavenumber range from 4500 to 500 cm^−1^ with a resolution of 4 cm^−1^ in absorbance mode.

#### 2.3.2. ^1^H NMR

Proton nuclear magnetic resonance (^1^H NMR) spectra of the prepared bio-based prepolymers were obtained with the use of a Bruker spectrometer. The operating frequency was 400 MHz for protons. The ca. 10% *w*/*v* solutions of the prepolymers and polyols were prepared in a DMSO-d_6_ solvent at ambient temperature. The simulation and iteration of the spectra were carried out using Bruker Avance III HD TopSpin software v.4, number of scans (ns): 16; delays (D1): 1 s, (D16): 0.0002 s.

#### 2.3.3. Rheological Measurement

Rheological tests were carried out using a Brookfield R/S+CPS rheometer with a cone-plate geometry (C50-2 cone). Prepolymers obtained from ether polyol and ester polyol containing ca. 6% or 8% of free isocyanate groups were tested. The samples were tested at the following temperatures: 60 °C, 70 °C and 80 °C. Measurements were made at a fixed and controlled shear rate using Rheo3000 software version 1.2.1377.1.

The following program was applied:

- increasing shear rate from 0 to 300 s^−1^ in 120 s,
- constant shear rate of 300 s^−1^ for 120 s,
- decreasing shear rate from 300 to 0 s^−1^ in 120 s.

Rheological models were proposed that best describe the rheological behavior of fluids. The three most commonly used models were distinguished: Newton’s, Herschel-Bulkley’s, and Ostwald-de Waele’s. They are characterized below.

The Herschel-Bulkley model is the simplest model of flow curves of nonlinear plastic-viscous fluids. The model is expressed in the Equation (1):

(1) $$y=k_{1}+k_{2}x^{k_{3}}$$

The Newton model is characterized by ideal fluids that show a linear relationship between the shear stress and shear rate. This model is described by the Equation (2):

(2) $$y=k_{2}x$$

Ostwald-de Waele describes shear-thinning fluids without a yield point. This model is expressed in the Equation (3):

(3) $$y=k_{2}x^{k_{3}}$$

where:

*x*—shear rate [s^−1^]
*y*—shear stress [Pa]
*k*_1_—yield stress [Pa]
*k*_2_—consistency index [-]
*k*_3_—flow behavior index [-]

#### 2.3.4. Thermogravimetry (TGA)

The analysis of monomers and prepolymers was performed using a Perkin Elmer TGA Pyris 1. The test was carried out in the temperature range of 50–600 °C under nitrogen atmosphere flow rate of 20 mL/min, for samples weighing up to approx. 5 mg. The heating rate during the run was 20 °C/min.

## 3. Results and Discussion

### 3.1. Chemical Strucutre

#### 3.1.1. Fourier-Transform Infrared Spectroscopy

**Figure 1:**
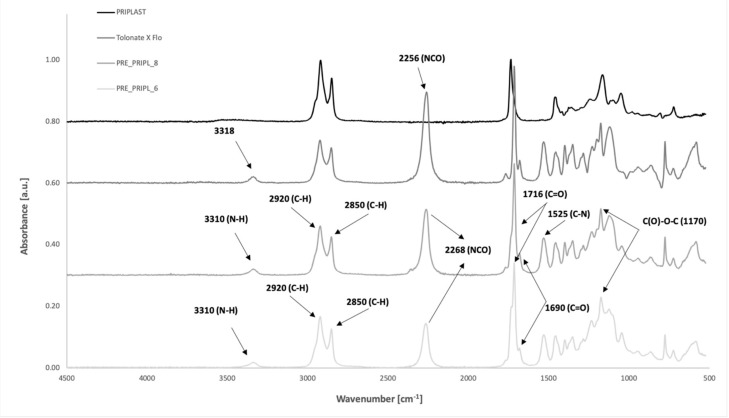
FTIR spectra of ester-urethane prepolymers and monomers.

**Figure 2:**
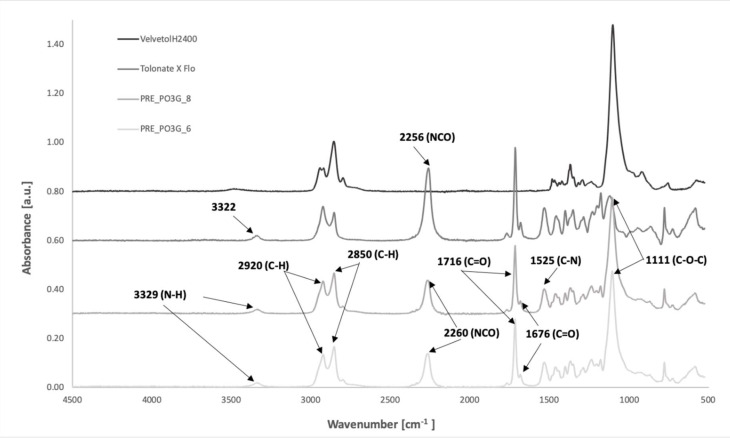
FTIR spectra of ether-urethane prepolymers and monomers.

FTIR is a useful method for determining the chemical composition of materials. The IR spectrum is used for identification. In this study, the qualitative and quantitative composition of the product was not determined, but mainly the spectrum obtained was compared with that of a standard sample. In the first step of the research, prepolymers PRE_PRIPL_6, PRE_PRIPL_8, PRE_PO3G_6, and PRE_PO3G_8 were synthesized. Spectra of obtained ether and ester-urethane prepolymers, and also monomers which were used for their preparation are presented in Figure 1 and Figure 2.

Figure 1 shows FTIR spectra for polyester polyol, partially bio-based isocyanate and prepolymers with ca. 6% and 8% of unreacted NCO groups. For each sample, characteristic functional groups were identified. Spectrum analysis of monomers and prepolymers allows to confirm the appearance of new bands related to the course of reaction between polyols and isocyanates. The FTIR spectra for all prepolymers (PRE_PO3G_6, PRE_PO3G_8, PRE_PRIPL_6 and PRE_PRIPL_8) are similar considering the presence of urethane and isocyanate groups, but differ in the intensity and location of the individual absorption peaks of the polyols. The formation of a urethane group characteristic for urethanes prepolymers and polyurethanes is confirmed by the band corresponding to stretching vibrations of -NH group of urethane group at wavenumber 3310 cm^−1^. Furthermore, bands were observed at a wavenumber between 2300–2200 cm^−1^, originating from stretching vibrations of the isocyanate group, confirmed by the Tolonate X Flo 100 spectrum [22]. It means that the excess of isocyanates was used and unreacted isocyanate groups, which are essential for the reaction with the chain extender, were still present at the terminal end of the prepolymer chain. Moreover, changes related to the band intensity coming from stretching vibrations of NCO groups were observed. An increase in the content of free isocyanate groups (%) in the prepolymer resulted in an increase in the area of this band, which confirms an increase in the number of these groups in the structure of the synthesized urethane prepolymer. Moreover, in the range of 1525–1530 cm^−1^ we observed a vibration stretching of the C-N bond and bending vibrations of -NH bonds in the urethane moiety [1]. Nevertheless, the greatest changes were observed in the band corresponding to valence vibrations of the NCO group. These vibrations, depending on the synthesis duration, differ in intensity and the area under the peak. Characteristic vibrations of the unbound C=O group of the urethane bond (1716 cm^−1^) and a band at the value of 1690 cm^−1^ were assigned [23]. At the region of carbonyl group vibration, derived from urethane linkage, also occurs the vibration of ester-based carbonyl characteristic for polyester polyol moiety. The band with a wavenumber of 1250 cm^−1^ indicates the presence of ester bonds present in Priplast. We also observe stretching vibrations of C-H bonds in the range of 2800–3000 cm^−1^ [23]. Asymmetric bending vibrations for the C-H bonds in CH_2_ group occur at a wavenumber of 1460 cm^−1^, while symmetrical vibrations are observed at the value of 1378 cm^−1^ [1]. It is observed that absorption bands of ester-urethane prepolymer with ca. 8% content of unreacted NCO groups (PRE_PRIPL_8) were analogous to those of PRE_PRIPL_6 prepolymer. The observed differences result from the change in the concentration of the unbound isocyanate groups and hence the differences in the intensity and area of the absorption bands of the isocyanate group. Vibrations of the unbound C=O group (1714 cm^−1^) and the bands at the value of 1690 cm^−1^ of PRE_PRIPL_8 sample are also characterized by higher intensity than in PRE_PRIPL_6 sample [24].

In Figure 2 spectra of ether-urethane prepolymers, polyether polyol and partially bio-based isocyanate were presented. For prepolymers PRE_PO3G_6 and PRE_PO3G_8, characteristic vibrations of the N-H bond, C-N bond, and C=O bond of the urethane group were also observed. The difference between ester and ether-urethane prepolymers is because the stretching vibrations of the carbonyl group was assigned as a double band at the value of 1734-1690 cm^−1^ [25]. The valence vibration of the NCO group was assigned at 2270 cm^−1^. Moreover, stretching vibrations were observed for the C-O-C group at a wavenumber of 1111 cm^−1^ for the polyol ether group present in PO3G [26]. The wavenumbers of C-H bond in CH_2_ group for stretching and bending vibration occurred at the range of 2800–3000 cm^−1^ and 1378–1460 cm^−1^, respectively.

#### 3.1.2. Proton Nuclear Magnetic Resonance

**Figure 3:**
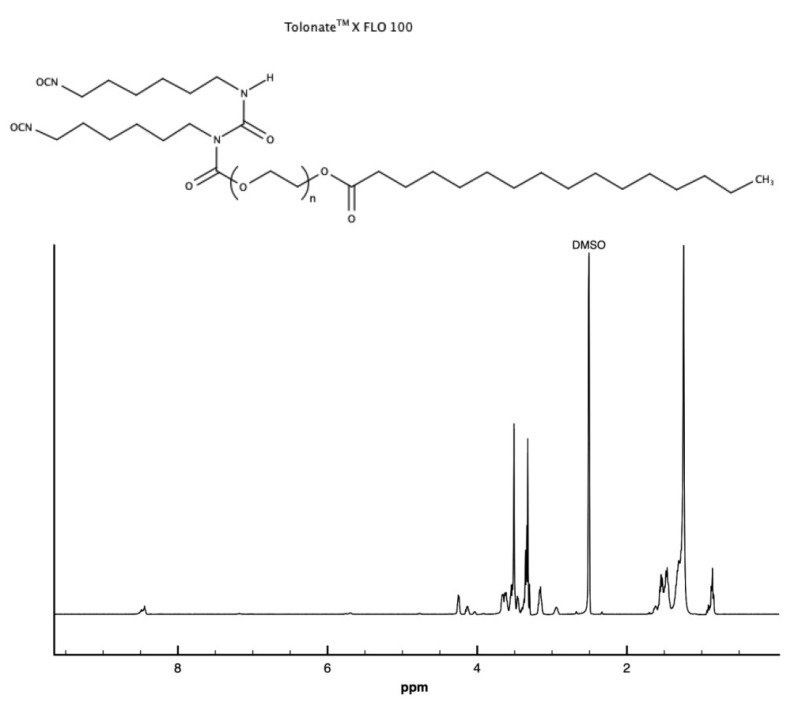
^1^H NMR spectra of Tolonate X FLO 100 sample.

**Figure 4:**
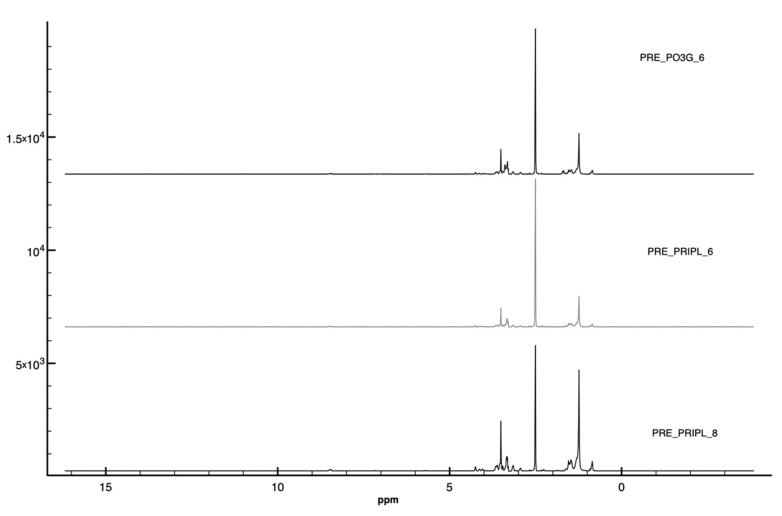
^1^H NMR spectra of PRE_PRIPL_6, PRE_PRIPL_8 and PRE_PO3G_6 prepolymers.

Figure 3 and Figure 4 present the spectra of Tolonate X FLO 100 and of the obtained ester-urethane and ether-urethane prepolymers.

The exact chemical formula of Tolonate X FLO diisocyanate was not disclosed by the manufacturer, however its structure was analyzed by a group of researchers from the University of Montpellier [21]. The NMR spectrum in Figure 3 shows several intense signals at 3.5 ppm, 2.5 ppm, 1.2 ppm. The signal at 3.5 ppm corresponds to the poly(ethylene glycol) chain. Protons of -CH_2_ groups, which are located inside the aliphatic chain of bio-diisocyanate, and protons of -CH_3_ group were observed in the range of 0.8–1.7 ppm. Meanwhile, the signal at 2.5 ppm was assigned to the protons of the solvent used (CD_3_)_2_SO (dDMSO). Based on the publication of Morales-Cerrada et al. it was concluded that the bio-based part of Tolonate X Flo is the palmitic acid moiety, while the poly(ethylene glycol) and chains containing isocyanate groups are derived from petroleum-based substances (ethylene glycol and hexamethylene diisocyanate) [21].

Figure 4 presents a spectrum comparison for polyester-urethane and polyether-urethane prepolymers. Based on the analysis of ^1^H NMR spectra, it was found that a reaction occurred between the -NCO group of the diisocyanate and the -OH group of the polyol used because a signal appeared in the range of the chemical shift of 8.52–8.42 ppm, characteristic of the protons in the -NH group of the urethane group [27]. The low intensity of this signal may be due to the unusual structure of the isocyanate, which does not contain aromatic rings that are present in typical isocyanates used for polyurethane synthesis. In the range of 3.69–3.58 ppm, numerous signals were observed associated with protons of the -CH_2_ groups which are linked to the oxygen atom of the ester group. In contrast, protons linked to the oxygen atom of the urethane group and the nitrogen atom of the urethane group were located at 3.57–3.1 ppm. At a shift value of 2.98–2.8, protons were observed in the immediate vicinity of the isocyanate group. Meanwhile, protons of -CH_2_ groups, which are located inside the aliphatic chain of bio-diisocyanate, bio-polyol and protons of -CH_3_ group were observed at a shift range of 2.98–0.8 ppm.

### 3.2. Processing Properties

#### 3.2.1. Rheological Measurement

Determining processing properties of prepolymers and polymers is necessary for the proper conduct of technological processes. The properties are assessed by adjusting the flows to the conditions in the processing tools [12]. Rheological measurements are used in many industries, including process engineering, bioengineering, or soil mechanics [28]. The combination of rheological studies and the structure and thermal properties of the prepolymer used allows for the best possible adjustment of the processing conditions.

The main parameter determined in rheological tests is viscosity. Viscosity describes resistance that occurs in a material as some layers move relative to others.

**Figure 5:**
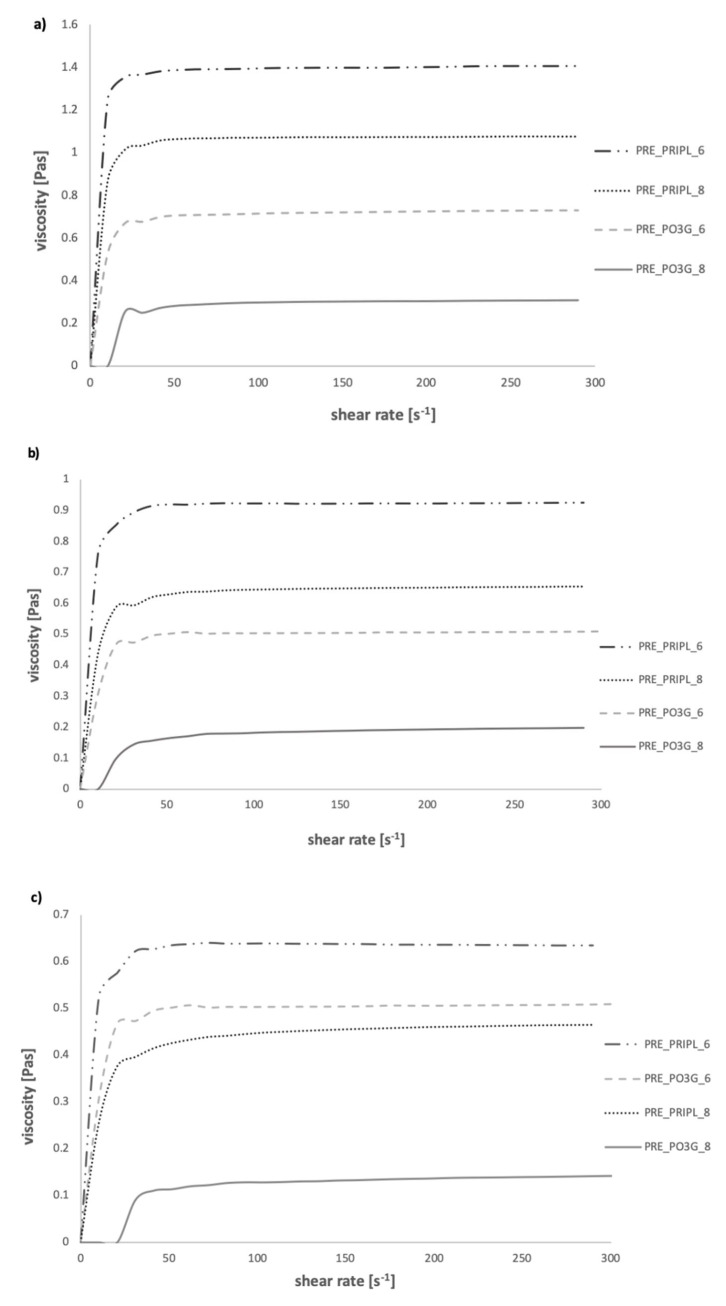
Viscosity curves of the synthesized bio−based polyols at (**a**) 60 °C, (**b**) 70 °C, (**c**) 80 °C.

**Figure 6:**
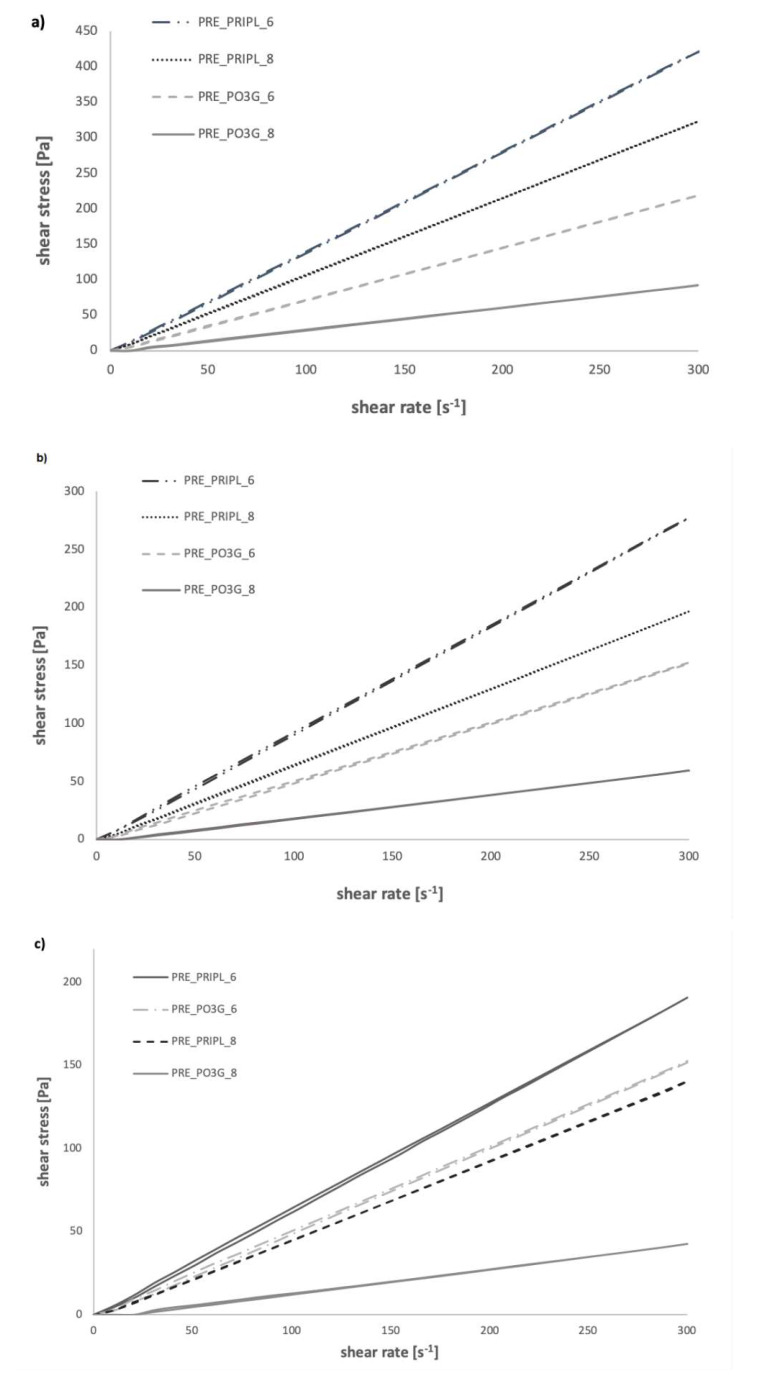
Flow curves of the synthesized bio−based polyols at (**a**) 60 °C, (**b**) 70 °C, (**c**) 80 °C.

The rheological analysis provided information on the dependence of viscosity in the function of shear rate (Figure 5), and on the dependence of shear stress in the function of shear rate (Figure 6). The measurements were carried out at 60 °C, 70 °C, and 80 °C, and the share rate was at the range from 0 to 300 s^−1^.

#### 3.2.2. Viscosity Curves of Prepolymers

Depending on the polyol which was used, the studied prepolymers show significant differences in viscosity curves and flow curves. Based on Figure 5, it was found that ether-urethane prepolymers have lower viscosities than ester-urethane prepolymers. In practice, ether-urethane prepolymer is a better processing material. Lower viscosity of the raw material is more advantageous in the preparation of polyurethane materials as it facilitates introduction of the filler and as a result it is possible to ensure a higher homogenization of the system. The course of the viscosity curve shows that both prepolymers belong to shear-thickening liquids and dilatation fluids, which means that their viscosity increases with the increasing shear rate [29]. For prepolymers with a higher content of free isocyanate groups, greater differences in viscosity are evident: at T = 60 °C for ca. 8% of unreacted NCO group the viscosity is about 0.8, and for NCO = 6% it is 0.7.

#### 3.2.3. Flow Curves of Prepolymers

In Figure 6, it is observed that the ascending curve for prepolymers with ca. 8% of unreacted NCO groups is above the descending curve, which means that the liquids are thixotropic. Moreover, at a temperature of 60 °C, it was observed that the curves coincide in both directions, which means that the fluid is rheologically stable. On the other hand, at T = 70 °C and T = 80 °C, slight hysteresis loops related to the destruction of the structure were recorded (Figure 6). In case of flow curves for prepolymers with ca. 6% content of unbound NCO groups, no hysteresis loops were noticed, which means that with shear forces acting, the structure was not destroyed. For PRE_PO3G_6 and PRE_PRIPL_6 prepolymers at T = 60 °C, the descending curve was observed above the ascending curve, which means that the liquid is anti-thixotropic. As for the viscosity curves and flow curves recorded at temperatures: 70 and 80 °C, their course is similar to that of the prepolymers tested at T = 60 °C. An increase in temperature reduces the differences in viscosity of the tested prepolymers. The differences in the maximum viscosity are as follows: at T = 60 °C–0.8 Pa∙s, at T = 70 °C–0.4 Pa∙s, and at T = 80 °C–0.1 Pa∙s. No hysteresis loops were observed for the flow curves at T = 70 °C. A difference in the course of the flow curve was observed at T = 80 °C. The ascending curve was above the descending curve, which means that the liquid is thixotropic. In addition, a slight hysteresis loop appears, which indicates that the structure is damaged by shear forces.

For all the temperatures, it was found that the dynamic viscosity coefficient increases with the increase in the shear rate, which allowed us to classify the fluids as shear thickened, and dilatation.

Based on the data in Figure 5, we could conclude that ester-urethane prepolymer with ca. 6% of unbound NCO groups was characterized by the highest viscosity. On the other hand, the lowest viscosities were displayed by ether-urethane prepolymer with 8% content of free NCO group. It was also observed that prepolymers with ca. 6% free NCO group were characterized by higher viscosity than prepolymers with higher content of NCO groups. These results show a significant influence of the content of free isocyanate groups, and of the substrate used on the viscosity of the systems. Systems with lower viscosities are more advantageous to use, especially in industry. We observed a characteristic decrease in viscosity with increasing temperature for all the prepolymers studied.

Table 2 shows a comparison of the best-fitted models with constants for the obtained prepolymers. The R parameter value indicates the degree of adjustment to a given model. All the models were determined using Rheo3000 software. Most of the samples were characterized with the Hershel-Bulkley rheological model, only two of the samples were described by another model. The sample of PRE_PRIPL_6 at 80 °C was described by the Newton model, and the sample of PRE_PRIPL_8 at 60 °C by the Ostwald model. The formulas for each model are presented below (Table 2).

### 3.3. Thermal Stability

#### Thermogravimetry

Thermogravimetric analysis is used in the thermal analysis of materials. Characterization of samples is performed by measuring changes in sample mass as a function of temperature.

The thermal properties of prepolymers and polyurethanes greatly depend on the substrates used in their synthesis. Due to the emission of gaseous products, their thermal degradation is a very complex process. Thermogravimetric curves provide information on the segmental structure of polyurethane materials and allow the determination of the percentage variation of the sample mass as a function of temperature.

**Figure 7:**
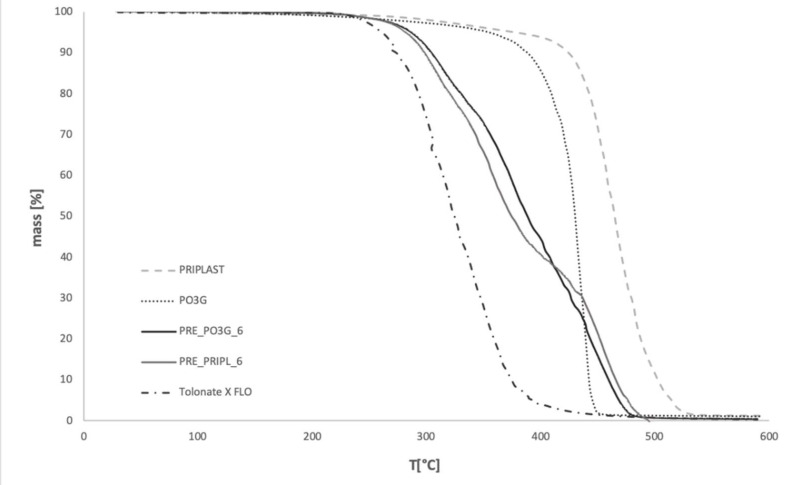
Dependence of TG in function of temperature for monomers and the obtained bio-based prepolymers with NCO ca. 6%.

**Figure 8:**
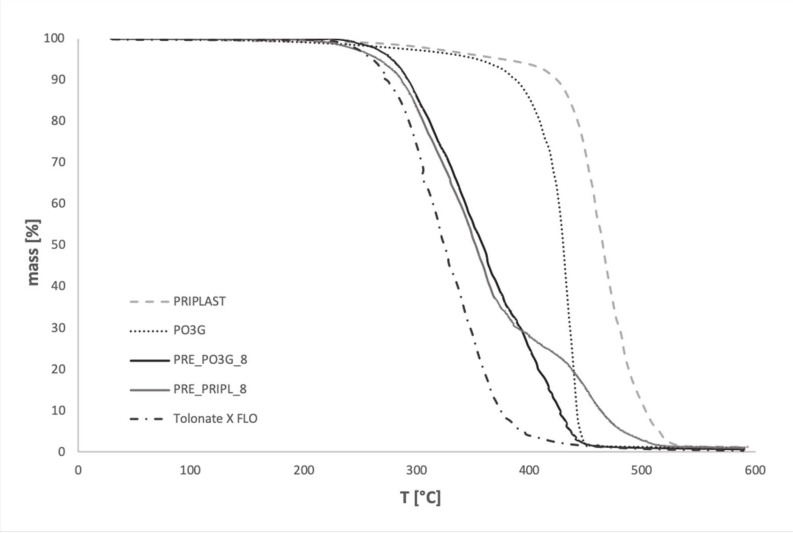
Dependence of TG in function of temperature for monomers and the obtained bio-based prepolymers with NCO ca. 8%.

**Figure 9:**
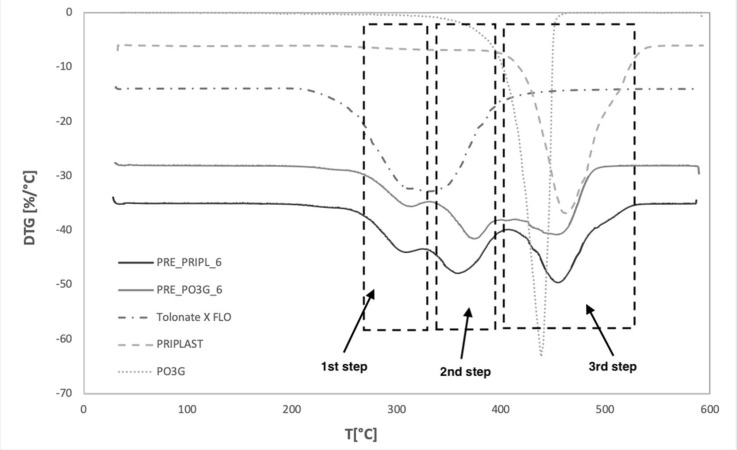
Dependence of DTG in the function of temperature for monomers and the obtained bio−based prepolymers with NCO ca. 6%.

**Figure 10:**
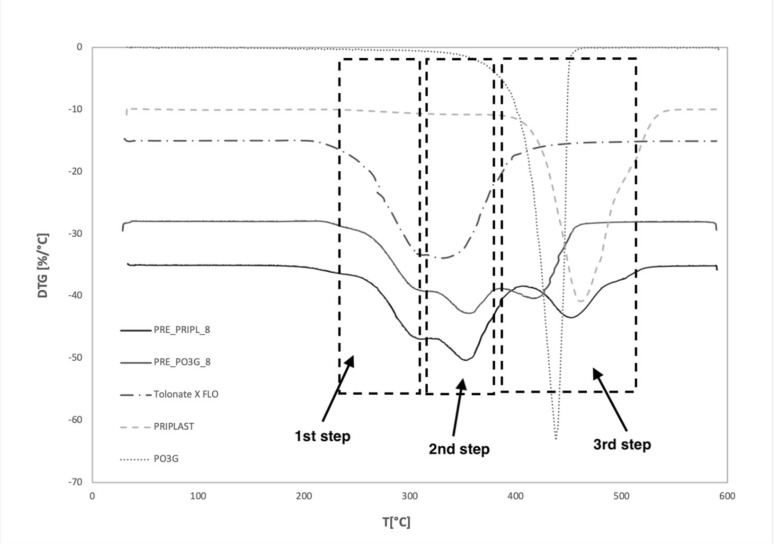
Dependence of DTG in the function of temperature for monomers and the obtained bio−based prepolymers with NCO ca. 8%.

Thermal decomposition of urethane prepolymers and polyurethane materials occurs in several stages. Thermogravimetric curves of the synthesized urethane prepolymers are shown in Figure 7, Figure 8, Figure 9 and Figure 10. The DTG and TG curves show that the decomposition of prepolymers displays a three-step-mass loss tendency. Thermogravimetric analysis of monomers clearly indicated which step in the decomposition of the prepolymers originated from each component.

Usually, during the decomposition of polyurethane materials, the first step is related to the decomposition of hard segments which consist of diisocyanate and low molecular weight chain extender [24,25]. In the case of urethane prepolymers, the first stage is related to the dissociation of unreacted diisocyanate. This stage is similar for all four urethane prepolymers, is associated with the degradation of the unreacted isocyanate, and occurs in the temperature range 233–321. The second stage occurs in the temperature range 350–380 and is associated with the dissociation of urethane group. The third stage of degradation is related to the decomposition of polyol [24]. As seen in Figure 7 and Figure 8, degradation of the ester-urethane prepolymer occurs at a higher temperature than that for ether-urethane prepolymer, which is related to a greater proportion of hard segments that degrade first.

Analyzing the thermal stability of used monomers, Table 3 shows a dependence in which the loss of 5% by weight of ester polyol occurs at a higher temperature (376.7 °C) than that for ether polyol (353.9 °C). This is due to the branched structure of ester polyol and its higher molecular weight. The work of J. Amado also confirmed higher thermal stability of polyester polyol than of polyether polyol. This is due to a general relationship in which an ether bond degrades at a lower temperature than the ester bond [30]. Table 3 presents a summary of temperatures at which the weight loss for selected monomers and bio-based urethane prepolymers was 5, 10, 50 and 90%, respectively. The last column of Table 3 contains residue amounts after combustion at 550 °C.

## 4. Conclusions

To sum up, we studied four novel bio-based isocyanate-terminated polyurethane prepolymers obtained with the use of two types of polyols: polyether and polyester, both of renewable origin, in the reaction with excess bio-based isocyanate (with 32% content of green carbon). Prepolymers with the content of ca. 6% and 8% unreacted isocyanate group were obtained. The chemical formula of the obtained prepolymers was confirmed by FTIR and NMR analysis. The two types of prepolymers are different in regard to their rheological properties, irrespective of NCO content. Polyester-based prepolymers were characterized by over 200% higher viscosity at the temperature of 60 °C than polyether-based prepolymers. In general, viscosity depends on temperature, and at a higher temperature lower differences in viscosity between both types of prepolymers were observed.

In general, it was demonstrated that both prepolymers degraded in three steps, similar to polyurethane materials. Their thermal stability is closely related to the type of bio-based polyols which were used for the pre-polymerization process. The beginning of thermal degradation expressed as T_5%_ is about 10 °C higher for urethane prepolymers based on PO3G polyol.

A proper understanding of the influence of the structure on rheological properties of substances allows for designing an optimal model of the materials manufacturing process. Obtained bio-based prepolymers have a high content of green carbon and because of their good rheological characteristics and thermal stability, they can be processed, for example, by using an industrial grades technique such as RIM (reactive injection molding technique). It is also possible to obtain foamed or cast materials with the use of prepared urethane prepolymers. Polyurethanes obtained with the use of bio-based urethane prepolymers can exhibit many important functional properties such as high elasticity, good abrasion resistance and good vibration damping properties. The properties of these materials can be freely modified by appropriate selection of components, synthesis conditions, and ratios of individual substrates.

## Figures and Tables

**Table 1:** NCO content.

Composition of Sample	Calculated Percentage of Unreacted-NCO Groups	Sample Code	Determined Percentage of Unreacted Isocyanate Groups [%]
Tolonate X FLO + Velvetol H2400	6%	PRE_PO3G_6	5.22 ± 0.03
Tolonate X FLO + PRIPLAST 3294TM	6%	PRE_PRIPL_6	5.51 ± 0.14
Tolonate X FLO + Velvetol H2400	8%	PRE_PO3G_8	7.28 ± 0.03
Tolonate X FLO + PRIPLAST 3294TM	8%	PRE_PRIPL_8	8.42 ± 0.08

**Table 2:** Rheological model of the obtained prepolymers.

Sample Code	Equation	k_1_	k_2_	k_3_	R
PRE_PO3G_6 (60 °C)	y = 0.65x^1.02^	0	0.65	1.02	0.9999
PRE_PO3G_6 (70 °C)	y = 0.47x^1.01^	0	0.47	1.01	0.9999
PRE_PO3G_6 (80 °C)	y = 0.38x^0.99^	0	0.38	0.99	0.9998
PRE_PO3G_8 (60 °C)	y = 0.25x^1.04^	0	0.25	1.04	0.9998
PRE_PO3G_8 (70 °C)	y = 0.12x^1.09^	0	0.12	1.09	0.9996
PRE_PO3G_8 (80 °C)	y = 0.08x^1.10^	0	0.08	1.10	0.9998
PRE_PRIPL_6 (60 °C)	y = 1.34x^1.01^	0	1.34	1.01	0.9999
PRE_PRIPL_6 (70 °C)	y = 0.90x^1.01^	0	0.90	1.01	0.9999
* PRE_PRIPL_6 (80 °C)	y = 0.65x	a	0.65	-	0.9999
** PRE_PRIPL_8 (60 °C)	y = x^1.01^	0	1	1.01	0.9999
PRE_PRIPL_8 (70 °C)	y = 0.59x^1.02^	0	0.59	1.02	0.9999
PRE_PRIPL_8 (80 °C)	y = 0.37x^1.04^	0	0.37	1.04	0.9998

* The best rheological model–Newton; ** Ostwald’s best model; a—not applicable.

**Table 3:** Thermal decomposition characteristics of the obtained bio-based materials.

Sample Code	T_5_ [°C]	T_10_ [°C]	T_50_ [°C]	T_90_ [°C]	m_T550 [°C]_ [%]
PO3G	353.9	387.9	429.9	441.9	1.1
PRIPL	376.7	424.1	464.8	502.7	1.3
FLO	261.5	274.2	324.7	374.5	0.45
PRE_PRIPL_6	280.0	297.8	372.3	475.1	1.1
PRE_PO3G_6	286.4	303.9	388.3	459.4	0.4
PRE_PRIPL_8	266.9	286.2	352.4	463.1	1.24
PRE_PO3G_8	277.9	292.2	358.4	426.9	0.8

T_5_ is the temperature of 5% mass loss, T_10_ is the temperature of 10% mass loss, T_50_ is the temperature of 50% mass loss and T_90_ is the temperature of 90% mass loss.

## Data Availability

Not applicable.

## Supplementary Materials

The following are available online at https://www.mdpi.com/article/10.3390/ijms222212207/s1.
