# Supplementary material for: Eco-Friendly Ether and Ester-Urethane Prepolymer: Structure, Processing and Properties
Source: Int J Mol Sci. 2021 Nov 11;22(22):12207. doi: 10.3390/ijms222212207 (PMC8625470; doi:10.3390/ijms222212207)
Supplement: Supplementary file 1 [file ijms-22-12207-s001.zip › ijms-1404643-supplementary.pdf]

Supplementary material

ECO-FRIENDLY ETHER AND ESTER-URETHANE PREPOLYMER: STRUCTURE, PROCESSING AND PROPERTIES

Joanna Niesiobędzka [1], Ewa Głowińska [1,*] and Janusz Datta [1]

[1] Gdańsk University of Technology, Faculty of Chemistry, Department of Polymer Technology, G. Narutowicza St. 11/12, 80-233 Gdańsk
* Correspondence: ewa.glowinska@pg.edu.pl; Tel.: +48 (58) 347 15 87

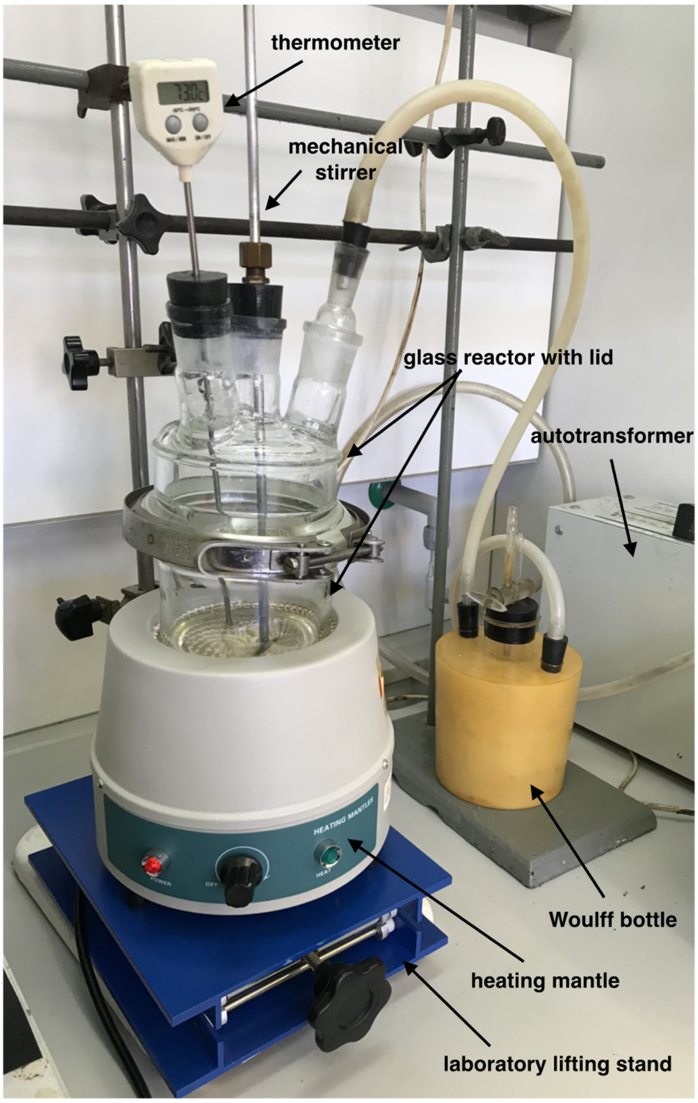

Figure S1. Picture of laboratory apparatus for the synthesis of urethane prepolymers.
